# A new method based on SNP of nrDNA-ITS to identify *Saccharum spontaneum* and its progeny in the genus *Saccharum*

**DOI:** 10.1371/journal.pone.0197458

**Published:** 2018-05-16

**Authors:** Shan Yang, Xueting Li, Fei Huang, Yongji Huang, Xinlong Liu, Jiayun Wu, Qinnan Wang, Zuhu Deng, Rukai Chen, Muqing Zhang

**Affiliations:** 1 National Engineering Research Center for Sugarcane, Fujian Agriculture and Forestry University, Fuzhou, China; 2 Yunnan Key Laboratory of Sugarcane Genetic Improvement, Sugarcane Research Institute, Yunnan Academy of Agricultural Sciences, Kaiyuan, China; 3 Guangdong Provincial Bioengineering Institute, Guangzhou Sugarcane Industry Research Institute, Guangzhou, China; 4 Guangxi Collaborative Innovation Center of Sugar Industries, Guangxi University, Nanning, China; National Cheng Kung University, TAIWAN

## Abstract

The identification of germplasm resources is an important aspect of sugarcane breeding. The aim of this study was to introduce a new method for identifying *Saccharum spontaneum* and its progeny. First, we cloned and sequenced nuclear ribosomal DNA internal transcribed spacer (nrDNA-ITS) sequences from 20 *Saccharum* germplasms. Analysis of these nrDNA-ITS sequences showed a stable mutation at base 89. Primers (FO13, RO13, FI16, and RI16) were then designed for tetra-primer amplification refractory mutation system (ARMS) PCR based on mutations at base 89 of the nrDNA-ITS sequence. An additional 71 *Saccharum* germplasms were identified using this tetra-primer ARMS PCR method, which confirmed that the method using the described primers successfully identified *Saccharum spontaneum* and progeny. These results may help improve the efficiency of modern molecular breeding of sugarcane and lay a foundation for identification of sugarcane germplasms and the relationships among them.

## Introduction

Sugarcane is an important sugar and energy crop worldwide. Sugarcane plants belong to the grass family Gramineae, genus *Saccharum*, and related plants in this family include *Miscanthus*, *Sclerostachya*, *Erianthus*, and *Narenga*. The *Saccharum* genus consists of six species, including *Saccharum officinarum* (2n = 80), *S*. *sinense* (2n = 112–120), *S*. *barberi* (2n = 82–124), *S*. *edule* (2n = 60, 70, 80), and two wild species, *S*. *robustum* (2n = 60–120) and *S*. *spontaneum* (2n = 40–128) [1]. *S*. *barberi* and *S*. *sinense* are secondary species derived from hybridization of *S*. *officinarum* and *S*. *spontaneum* [2]. Moreover, *S*. *sinense*, *S*. *barberi*, *S*. *robustum*, *S*. *spontaneum*, and *S*. *officinarum* are important parental resources in sugarcane breeding [3]. Sugarcane cultivars are multiple interspecific hybrids and highly heterogeneous, and almost all contain some *S*. *spontaneum* genetic material at levels up to 10% of all chromosomes [4].

Internal transcribed spacers (ITS) of nuclear ribosomal DNA (nrDNA) contain ITS1, 5.8s rDNA, and ITS2 [5]. In recent years, several features of nrDNA-ITS have made a useful tool for evaluating and analyzing evolutionary relationships at the subspecies level, including rich variances and rapid evolutionary rate, as well as simple PCR amplification and sequencing [6–8]. In view of these features, Yang et al. analyzed the nrDNA-ITS sequence characteristics of 19 *S*. *spontaneum* germplasms and 11 local sugarcane varieties [9]. The results showed that 11 *S*. *spontaneum* germplasms could be divided into several branches, and that local sugarcane varieties were closely related to *S*. *spontaneum*. Moreover, the ITS1 sequence could be used as a DNA barcode to further study the genetic diversity of *Saccharum* and related genera. Liu et al. analyzed differences among nrDNA-ITS sequences from 62 different multiple *S*. *spontaneum* materials and showed that 4 species had high variation, especially the nonuploid and decaploid population [10].

As a third-generation molecular marker, single nucleotide polymorphisms (SNPs) are highly stable and widely used for studies of crop molecular genetics [11]. Due to the limited availability of sugarcane genomic maps, research on SNPs in sugarcane lags behind that of rice, rapeseed, and other crops [12]. SNPs for many crops have been discovered through analysis of nrDNA-ITS sequences and in turn have served as valuable molecular markers to identify interspecies germplasms [13] that can contribute to strategies for molecular breeding of crops [14]. Tetra-primer amplification refractory mutation system PCR (tetra-primer ARMS PCR) is a derivative technique based on common PCR that can be specifically used to detect SNPs [15]. Tetra-primer ARMS PCR is rapid, simple, and economical. According to the SNP site, the tetra-primer ARMS PCR technique has been used to identify various germplasm genotypes in rice, wheat, capsicum, and other crops [16–18].

Based on previous studies on nrDNA-ITS in sugarcane germplasms, the tetra-primer ARMS PCR technique can be used to analyze genetic diversity and phylogenetic relationships in interspecific and intergenus samples [9, 10, 19]. However, studies exploring the identification and use of SNPs as molecular markers in *Saccharum* breeding have not been performed. As such, we cloned, sequenced, and analyzed nrDNA-ITS sequences of 20 *Saccharum* germplasms to identify a stable SNP. Based on the SNP site, primers were designed according to the principles of tetra-primer ARMS PCR. PCR of 71 materials was performed to identify the presence of *Saccharum spontaneum* genetic material. This study provides a foundation for improving the efficiency of modern molecular breeding of sugarcane and a molecular basis for identifying sugarcane germplasms.

## Materials and methods

### Plant materials

In this study, 20 clones were selected for nrDNA-ITS sequencing, including 5 *S*. *officinarum*, 5 *S*. *robustum*, and 15 different multiple *S*. *spontaneum* samples that contained octoploid, nonuploid, decaploid, dodecaploid, and tridecaploid *S*. *spontaneum* (Table 1). A total of 71 clones were selected for testing and analysis of the specificity of tetra-primer ARMS PCR primers that we developed and designed, including 5 *S*. *officinarum* and 3 F_1_ (*S*. *officinarum*×*S*. *spontaneum*), which all have similar morphology to *S*. *officinarum*, 6 *S*. *robustum*, 43 *S*. *spontaneum*, 3 *S*. *sinense*, 3 *S*. *barberi*, 3 sugarcane cultivars and 5 F_1_ (*S*. *officinarum*×*S*. *robustum*) materials (Table 2).

**Table 1:** Plant materials for cloning and sequencing of nrDNA-ITS sequences.

No.	Clone name	Species name	Ploidy	Number of chromosomes
1	Badila	*Saccharum officinarum*	octoploid	80
2	VN cattle cane	*Saccharum officinarum*	octoploid	80
3	Loethers	*Saccharum officinarum*	octoploid	80
4	Crystalina	*Saccharum officinarum*	octoploid	80
5	S. Cheribon	*Saccharum officinarum*	octoploid	80
6	57NG208	*Saccharum robustum*	octoploid	80
7	51NG63	*Saccharum robustum*	octoploid	80
8	NG77-004	*Saccharum robustum*	octoploid	80
9	28NG21	*Saccharum robustum*	octoploid	80
10	Daye	*Saccharum robustum*	octoploid	80
11	YN75-2-11	*Saccharum spontaneum*	octoploid	64
12	YN82-110	*Saccharum spontaneum*	octoploid	64
13	YN83-160	*Saccharum spontaneum*	octoploid	64
14	FJ89-1-1	*Saccharum spontaneum*	nonuploid	72
15	YN83-201	*Saccharum spontaneum*	nonuploid	72
16	YN82-44	*Saccharum spontaneum*	decaploid	80
17	YN83-171	*Saccharum spontaneum*	decaploid	80
18	GZ78-2-28	*Saccharum spontaneum*	dodecaploid	96
19	FJ88-1-13	*Saccharum spontaneum*	dodecaploid	96
20	FJ89-1-19	*Saccharum spontaneum*	tridecaploid	104

**Table 2:** Plant materials used for identifying genetic material from *Saccharum spontaneum* with tetra-primer ARMS PCR.

No.	Clone name	Species name	Ploidy	Number of chromosomes
1	Badila	*Saccharum officinarum*	octoploid	80
2	VN cattle cane	*Saccharum officinarum*	octoploid	80
3	Loethers	*Saccharum officinarum*	octoploid	80
4	Crystalina	*Saccharum officinarum*	octoploid	80
5	S. Cheribon	*Saccharum officinarum*	octoploid	80
6	Muckche	F_1_(*S*. *officinarum*×*S*. *spontaneum*)	octoploid	141–143
7	Baimeizhe	F_1_(*S*. *officinarum*×*S*. *spontaneum*)	octoploid	104–106
8	Cana Blanca	F_1_(*S*. *officinarum*×*S*. *spontaneum*)	octoploid	113–115
9	28NG21	*Saccharum robustum*	octoploid	80
10	51NG63	*Saccharum robustum*	octoploid	80
11	51NG3	*Saccharum robustum*	octoploid	80
12	Daye	*Saccharum robustum*	octoploid	80
13	57NG208	*Saccharum robustum*	octoploid	80
14	NG77-004	*Saccharum robustum*	octoploid	80
15	YN75-2-11	*Saccharum spontaneum*	octoploid	64
16	YN83-160	*Saccharum spontaneum*	octoploid	64
17	YN83-225	*Saccharum spontaneum*	octoploid	64
18	YN82-58	*Saccharum spontaneum*	octoploid	64
19	YN4	*Saccharum spontaneum*	octoploid	64
20	YN83-238	*Saccharum spontaneum*	octoploid	64
21	Vietnam-3	*Saccharum spontaneum*	octoploid	64
22	YN82-9	*Saccharum spontaneum*	octoploid	64
23	YN-mengzi	*Saccharum spontaneum*	octoploid	64
24	YN84-268	*Saccharum spontaneum*	octoploid	64
25	YN82-110	*Saccharum spontaneum*	octoploid	64
26	GZ78-1-11	*Saccharum spontaneum*	nonuploid	72
27	YN76-1-16	*Saccharum spontaneum*	nonuploid	72
28	FJ89-1-11	*Saccharum spontaneum*	nonuploid	72
29	YN82-50	*Saccharum spontaneum*	nonuploid	72
30	SC92-42	*Saccharum spontaneum*	nonuploid	72
31	FJ89-1-1	*Saccharum spontaneum*	nonuploid	72
32	YN83-201	*Saccharum spontaneum*	nonuploid	72
33	SC88-49	*Saccharum spontaneum*	decaploid	80
34	FJ89-1-21	*Saccharum spontaneum*	decaploid	80
35	YN76-1-24	*Saccharum spontaneum*	decaploid	80
36	YN75-2-35	*Saccharum spontaneum*	decaploid	80
37	SC79-2-16	*Saccharum spontaneum*	decaploid	80
38	SC79-1-26	*Saccharum spontaneum*	decaploid	80
39	YN83-171	*Saccharum spontaneum*	decaploid	80
40	Wenshan cane	*Saccharum sinense*	Unknown	Unknown
41	Uba	*Saccharum sinense*	Unknown	116–118
42	GD-sinense	*Saccharum sinense*	Unknown	Unknown
43	Nagans	*Saccharum barberi*	Unknown	Unknown
44	Pansahi	*Saccharum barberi*	Unknown	Unknown
45	Saretha	*Saccharum barberi*	Unknown	91–92
46	ROC10	Cultivars	Unknown	Unknown
47	ROC22	Cultivars	Unknown	Unknown
48	CP84-1198	Cultivars	Unknown	Unknown
49	FJ92-1-11	*Saccharum spontaneum*	decaploid	80
50	Heqing	*Saccharum spontaneum*	decaploid	80
51	YN75-2-35	*Saccharum spontaneum*	decaploid	80
52	GD-16	*Saccharum spontaneum*	decaploid	80
53	GD-60	*Saccharum spontaneum*	decaploid	80
54	GD-71	*Saccharum spontaneum*	decaploid	80
55	YN82-44	*Saccharum spontaneum*	decaploid	80
56	Ledong-1	*Saccharum spontaneum*	decaploid	80
57	Xundian	*Saccharum spontaneum*	decaploid	80
58	GZ78-1-5	*Saccharum spontaneum*	decaploid	80
59	GZ79-1-4	*Saccharum spontaneum*	decaploid	80
60	FJ88-1-13	*Saccharum spontaneum*	dodecaploid	96
61	GZ78-2-28	*Saccharum spontaneum*	dodecaploid	96
62	FJ-HuiAn	*Saccharum spontaneum*	dodecaploid	96
63	FJ89-1-16	*Saccharum spontaneum*	dodecaploid	96
64	GD-30	*Saccharum spontaneum*	dodecaploid	96
65	FJ89-1-18	*Saccharum spontaneum*	dodecaploid	96
66	FJ89-1-19	*Saccharum spontaneum*	tridecaploid	104
67	RL12-38-1	F_1_(*S*. *officinarum*×*S*. *robustum*)	Unknown	Unknown
68	RL12-38-5	F_1_(*S*. *officinarum*×*S*. *robustum*)	Unknown	Unknown
69	RL12-38-76	F_1_(*S*. *officinarum*×*S*. *robustum*)	Unknown	Unknown
70	RL12-38-81	F_1_(*S*. *officinarum*×*S*. *robustum*)	Unknown	Unknown
71	RL12-38-84	F_1_(*S*. *officinarum*×*S*. *robustum*)	Unknown	Unknown

### Reagents and materials

Takala Ex Taq^®^ polymerase, Takala LA Taq^®^ polymerase, PMD19-T vector, and *E*. *coli* DH5α competent cells were obtained from Takara Biotechnology Co., Ltd. (Dalian of China). Primers were synthesized by the Beijing Genomics Institute (Beijing, China).

### Genomic DNA extraction

Young leaves from different sugarcane species were collected and powdered after freezing in liquid nitrogen. Genomic DNA was extracted from the leaves using a traditional CTAB method that was performed according to Porebski et al. [20].

### Cloning and sequencing

The nrDNA-ITS sequences from 20 clones (Table 1) were amplified using the universal primers ITS1 and ITS4 (ITS1: TCCGTAGGTGAACCTGCGG; ITS4: TCCTCCGCTTATTGATATGC) [21]. The PCR reaction mixtures were prepared on ice (Table 3) and carried out in a thermal cycler (ABI, 9902, USA). The reaction sequences were as follows: pre-denaturation at 95°C for 5 min followed by 35 cycles of 95°C for 15 s, 54°C for 15 s, and 72°C for 10 s. A final extension was conducted at 72°C for 5 min. The PCR products were tested by 1.5% agarose gel electrophoresis and purified using an Omega EZNA gel extraction kit. The purified products were then cloned into a PMD19-T vector and transformed into *E*. *coli* DH5α competent cells. Recombinant clones were grown in LB medium supplemented with ampicillin (100 μg/mL). Five clones per sample were selected for sequencing by Sangon Biotech Co., Ltd. (Shanghai, China).

**Table 3:** nrDNA-ITS PCR reaction mixture.

Components	Volume (μL)
ddH_2_O	13.9
10× LA buffer (Mg^2+^ plus)	2.0
dNTP (2.5 mM each)	1.6
ITS1 (10 μM)	0.8
ITS4 (10 μM)	0.8
Template (gDNA; 50 ng/μL)	0.8
LA Taq (5 U/μL)	0.1
Total volume	20.0

### Sequence analysis

DNA sequence homology was estimated using a nucleotide BLAST tool in the NCBI database. All DNA sequences were analyzed by DNAMAN 6.0 and BioEdit 7.0.9.0 to obtain variable site information.

### Primer design

Optimized primers for PCR were designed according to the design principle of tetra-primer ARMS PCR primers. Specific reference to the design method for tetra-primer ARMS PCR primers is made in Medrano and de Oliveira [22].

### Tetra-primer ARMS PCR procedure

Tetra-primer ARMS PCR of 71 samples (Table 2) was performed using the primers FO13, RO13, FI16, and RI16 (FO13: GTTTTTGAACGCAAG TTGCGCCCGAGGC; RO13: AATTCGGGCGACGAAGCCACCCGATTCT; FI16: GCCGGCGCATCGGC CCTAAGGACCTAT; RI16: GAGCGGCTATGCGCTGCGGTGCTTCT). Tetra-primer ARMS PCR reaction mixtures were prepared on ice (Table 4) and carried out in a thermal cycler (ABI, 9902, USA). The reaction conditions were as follows: pre-denaturation at 95°C for 5 min, followed by 6 cycles of 95°C for 30 s, 78°C for 20 s for one cycle and descending by 1°C for each subsequent 20 s cycle, 72°C for 20 s; the reaction ended with 24 cycles of 95°C for 30 s, 71°C for 10 s, 72°C for 10 s, and a final extension at 72°C for 5 min. The PCR products were tested by 1.5% agarose gel electrophoresis.

**Table 4:** Tetra-primer ARMS PCR mixtures.

Components	Volume (μL)
ddH_2_O	1.4
2×GC buffer	10.0
dNTP (2.5 mM each)	2.4
Dimethylsulphoxide	0.8
FO13 (5 μM)	1.6
RO13 (5 μM)	1.2
FI16 (5 μM)	0.4
RI16 (5 μM)	1.6
Template (gDNA; 50 ng/μL)	0.4
Ex Taq (5 U/μL)	0.2
Total volume	20.0

## Results

### nrDNA-ITS PCR

**Fig 1:**
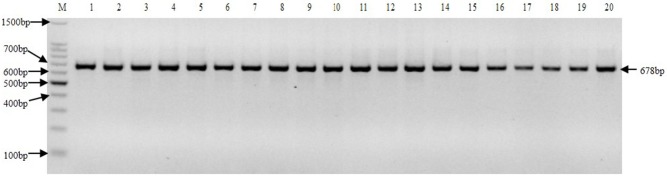
Electrophoretogram of nrDNA-ITS PCR products. M: 100 bp DNA ladder marker. Lanes 1–5: Badila, VN cattle cane, Loethers, Crystalina, Cheribon, respectively, belong to *S*. *officinarum*. Lanes 6–10: 57NG208, 51NG63, NG77-004, 28NG21, Daye, respectively, belong to *S*. *robustum*. Lanes 11–20: YN75-2-11, YN82-110, YN83-160, FJ89-1-1, YN83-201, YN82-44, YN83-171, GZ78-2-28, FJ88-1-13, FJ89-1-19, respectively, belong to *S*. *spontaneum*.

The nrDNA-ITS sequences of different samples were obtained by PCR with ITS1 and ITS4 primers. The nrDNA-ITS PCR product from each material tested appeared in the electrophoresis map as a single, intense 678 bp band (Fig 1).

### Sequence analysis

**Fig 2:**
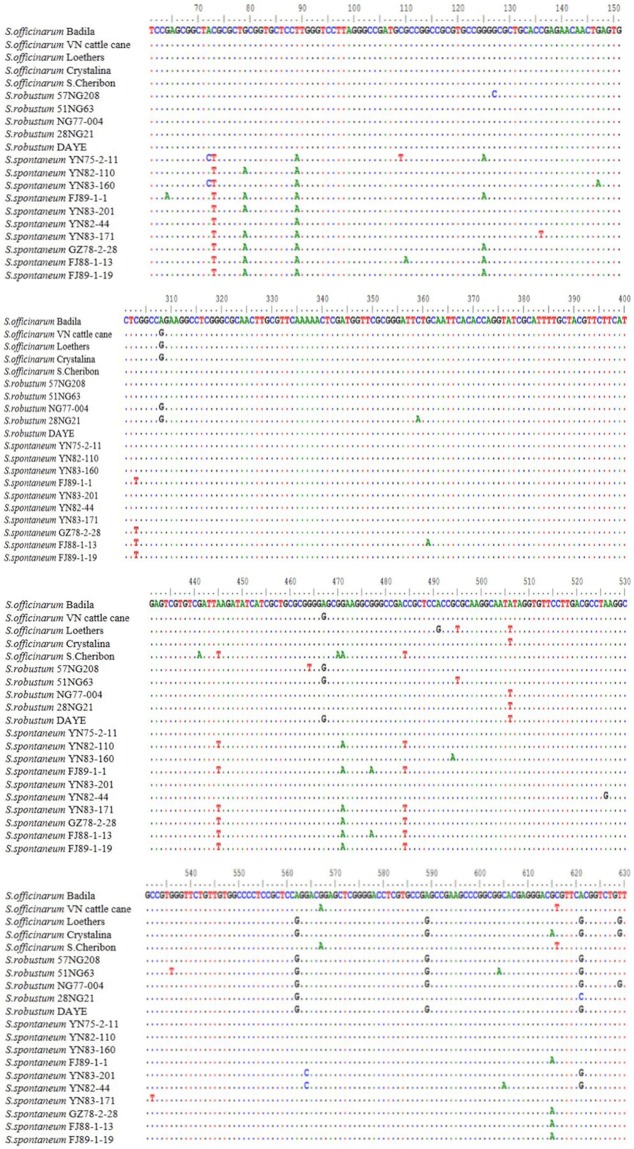
Analysis of nrDNA-ITS sequences. “······” represents sequences that are identical to that of Badila. Only base mutations are shown.

All of the clone sequences were analyzed using the BLAST tool in the NCBI database. The homology of all cloned sequences with other germplasm nrDNA-ITS sequences of sugarcane was >98%, which indicated that the clone sequences contained nrDNA-ITS sequences and were highly conserved. All clone sequences were analyzed using DNAMAN 6.0 and BioEdit 7.0.9.0 to obtain variable site information. At least five samples had mutations at bases 73, 79, 89, 125, 308, 445, 471, 484, 506, 562, 589, 615, and 621 (Fig 2). Among all mutations, those at base 73 and 89 were in a regular form because the mutation occurred only in *S*. *spontaneum* clones (Fig 2). However, only the mutation at base 89 was conserved for 10 *S*. *spontaneum* clones, which could be a good target region of tetra-primer ARMS PCR.

### Tetra-primer ARMS PCR

**Fig 3:**
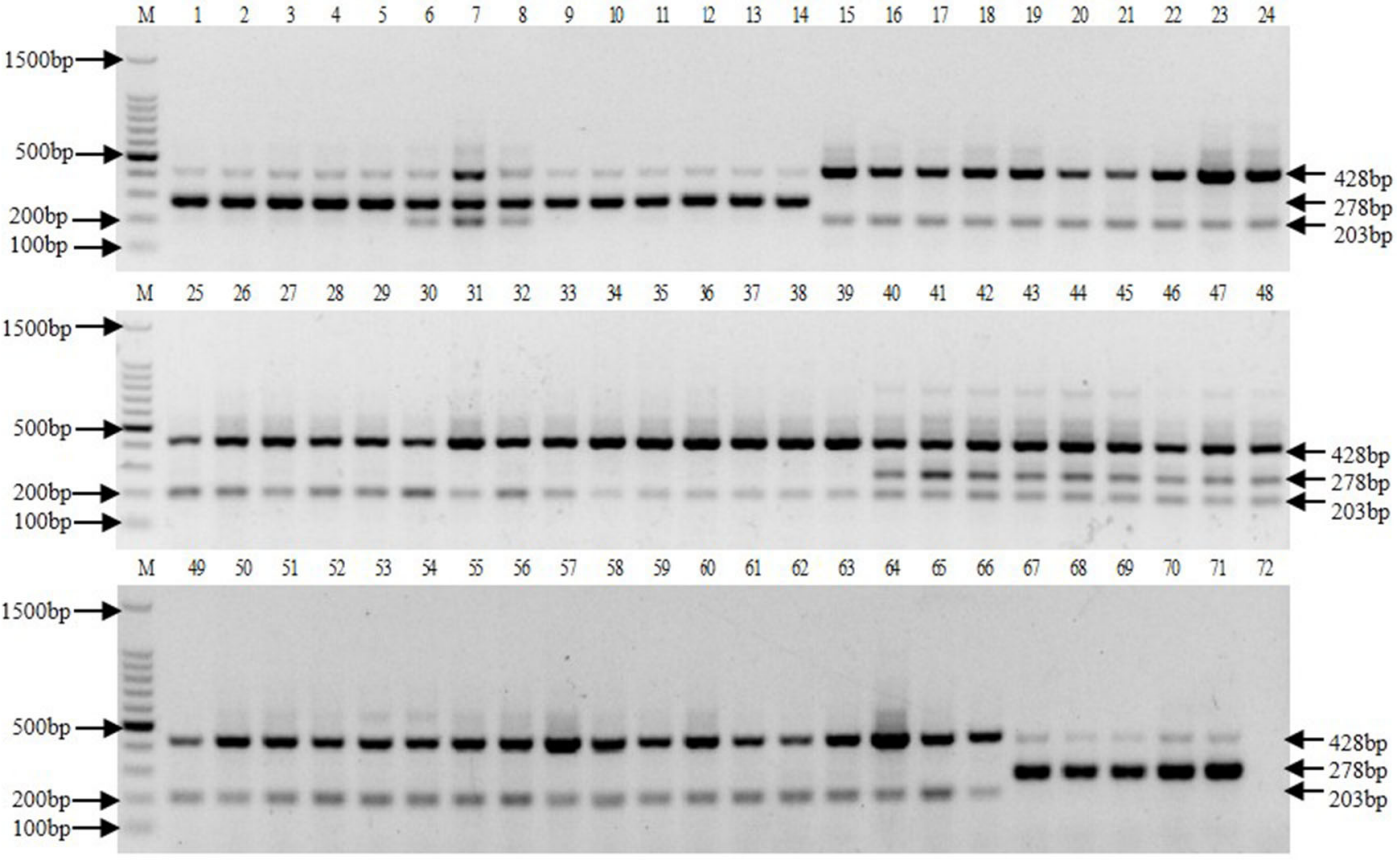
Electrophoretogram of tetra-primer ARMS PCR products. M: 100 bp DNA ladder marker. Lanes 1–5: Badila, VN cattle cane, Loethers, Crystalina, and S. Cheribon, respectively, are *S*. *officinarum*. Lanes 6–8: Muckche, Baimeizhe, and Canablanca, respectively, are F_1_ generations produced between *S*. *officinarum* and *S*. *spontaneum*. Lanes 9–14: 28NG21, 51NG63, 51NG3, Daye, 57NG208, and NG77-004, respectively, are *S*. *robustum*. Lanes 15–39: YN75-2-11, YN83-160, YN83-225, YN82-58, YN4, YN83-238, Vietnam-3, YN82-9, YN-mengzi, YN84-268, YN82-110, GZ78-1-11, YN76-1-16, FJ89-1-11, YN82-50, SC92-42, FJ89-1-1, YN83-201, SC88-49, FJ89-1-21, YN76-1-24, YN75-2-35, SC79-2-16, SC79-1-26, and YN83-171, respectively, are *S*. *spontaneum*. Lanes 40–42: Wenshan cane, Uba, and GD-sinense, respectively, are *S*. *sinense*. Lanes 43–45: Nagans, Pansahi, and Saretha, respectively, are *S*. *barberi*. 46–48: ROC10, ROC22, and CP84-1196, respectively, are cultivars. Lanes 49–66: FJ92-1-11, Heqing, YN75-2-35, GD-16, GD-60, GD-71, YN82-44, Ledong-1, Xundian, GZ78-1-5, GZ79-1-4, FJ88-1-13, GZ78-2-28, FJ-HuiAn, FJ89-1-16, GD-30, FJ89-1-18, and FJ89-1-19, respectively, are *S*. *spontaneum*. Lanes 67–71: RL12-38-1, RL12-38-5, RL12-38-76, RL12-38-81, and RL12-38-84, respectively, are F_1_ generations produced between *S*. *officinarum* and *S*. *robustum*. Lane 72: H_2_O.

A total of 71 *Saccharum* genus germplasm materials were amplified around base 89 using tetra-primer ARMS PCR. Tetra-primer ARMS PCR results showed a 428 bp common electrophoretic band in all materials tested, of which *S*. *spontaneum* had a 203 bp-specific electrophoretic band, whereas *S*. *officinarum* and *S*. *robustum* had a 278 bp-specific electrophoretic band (Fig 3). Considering the bands for ROC10, ROC22, and CP84-1196, we successfully identified the presence of *S*. *spontaneum* genetic material. RL12-38-1, RL12-38-5, RL12-38-76, RL12-38-81, and RL12-38-84 yielded two bands (428 bp and 278 bp), which indicated the absence of *S*. *spontaneum* genetic material. Moreover, analysis of electrophoretic bands allowed for the determination that Muckche, Baimeizhe, and Canablanca were F_1_ (*S*. *officinarum* × *S*. *spontaneum*) (Fig 3).

## Discussion

In this study, we found for the first time that base 89 was a stable base mutation in the nrDNA-ITS sequence of the *Saccharum* genus and was present in *S*. *spontaneum* genetic material. We thus designed primers for tetra-primer ARMS PCR based on this nrDNA-ITS sequence SNP. After optimization and identification, the primers FO13, RO13, FI16, and RI16 were found to be suitable for identification of *S*. *spontaneum* genetic material and its progeny. To the best of our knowledge, this is the first instance of the use of tetra-primer ARMS PCR to identify *S*. *spontaneum* genetic signatures in the *Saccharum* genus. These findings will be valuable for classifying sugarcane germplasms and will improve sugarcane hybridization breeding efficiency.

In angiosperms, this entire ITS region (ITS1+5.8S+ITS2) can be easily amplified using universal primers that recognize conserved coding regions to produce a 700 bp amplicon [23]. Here, a 678 bp fragment was amplified using the general primers ITS1 and ITS4 that cover the entire ITS sequence. This ITS sequence is not only widely used for germplasm classification and phylogeny analysis, but also for identification of germplasm resources in sample plants. Previous studies have suggested that this ITS sequence has higher conservation than the medium-height repetitive sequence and non-coded sequences, and that the mutation rate is relatively rapid compared with the coded gene sequence [24]. In allopolyploid plants, ITS sequence evolution is complex, such that ancestral ITS sequences can coexist in some offspring, but in other offspring may evolve in another direction [25, 26]. For example, in the polyploid plants of wheat, ancestral nrDNA-ITS sequences coexist in the offspring [27]. In this study, tetra-primer ARMS PCR results showed that the hybrid generation generated between *S*. *officinarum* and *S*. *spontaneum* yielded three bands (428 bp, 278 bp, 203 bp), the offspring generated between *S*. *officinarum* and *S*. *robustum* yielded two bands (428 bp, 278 bp), which respectively revealed parents’ traits. Therefore, we found that the ancestral nrDNA-ITS sequences could coexist in the offspring in the hybrid process of sugarcane. Our sequence analysis in this study indicated that the nrDNA-ITS sequence in sugarcane is relatively conserved. Moreover, the SNPs we identified were highly stable genetic markers. Therefore, the primers we developed for tetra-primer ARMS PCR could be used for accurate and reliable identification of *S*. *spontaneum* genetic material and progeny in the *Saccharum* genus.

The identification of *Saccharum* germplasm collections is mainly based on morphological observation, which can result in misclassification. In recent years, several molecular markers, such as SSR, AFLP, ISSR, and RAPD, have been applied for identification of *Saccharum* germplasms and progeny identification [28–31]. Many specific bands can be obtained from the progeny of hybrid offspring using these molecular markers, which contribute significantly to the separation and identification of sugarcane hybrids and germplasms. However, these molecular markers cannot intuitively identify sugarcane germplasms, as they produce multiple amplification bands that complicate interpretation and quantification. Of course, single-copy gene marker had been applied in phylogenetics in many plants with the development of sequencing technology [32, 33]. Compare with nrDNA-ITS sequence, the use of a single-copy gene mostly demands development of PCR primers specific for the taxonomic group of interest [34]. Moreover, this can result in the inclusion of paralogous copies in phylogenetic studies in the polyploid plans, resulting in wrong taxon relationships. To avoid this problem, the single-copy nuclear genes that occured mostly only with a single copy in the haploid genome might be preferable in phylogenetic analyses and identification of different species [35]. Therefore, selection of single-copy gene marker is an issue at present, especially in the polyploid plant. Because the sugarcane is an allopolyploids plant, its genome research is not yet complete and it is very difficult to obtain the haploid of sugarcane at present. Thus, selection of a single-copy gene as molecular marker is very difficult in sugarcane. In a more visual identification of sugarcane germplasm materials, Piperidis et al. used genomic in situ hybridization (GISH) to show that Kokea, Muntok Java, and Bourbonriet suriname were not *S*. *officinarum*, but instead were hybridized progeny of *S*. *officinarum* and *S*. *spontaneum* [36]. Moreover, many breeders believed that Muckche, Canablanca, and Baimeizhe were *S*. *officinarum* based on similar morphology. However, in 2016 Wang et al. identified 10 *S*. *officinarum* types using GISH technology and found that Muckche, Canablanca, and Baimeizhe were in fact hybridized progeny of *S*. *officinarum* and *S*. *spontaneum* [37]. These results are consistent with our study, which supports the reliability of the molecular markers we identified. Based on tetra-primer ARMS PCR results, we easily distinguished *S*. *spontaneum* and other *Saccharum* germplasm materials in this study. Moreover, tetra-primer ARMS PCR using the primers FO13, RO13, FI16, and RI16 to identify *S*. *spontaneum* and progeny is simpler and less time-consuming than GISH.

The breeding of a sugarcane variety typically requires approximately 10 years. Historically, the breeding process could not be accelerated because plants could not be selected early in the seedling stage due to a lack of molecular markers. The tetra-primer ARMS PCR technology developed in this study could have broad applications for sugarcane breeding in the future. This approach could address the problem of early selection and identify whether seedlings incorporate *S*. *spontaneum* genetic material. Germplasms from plants thought to include *S*. *spontaneum* as a predecessor could be identified using tetra-primer ARMS PCR technology to determine whether such plants are indeed hybridized progeny of *S*. *officinarum* and *S*. *spontaneum*.

## References

[1] Irvine JE. Saccharum species as horticultural classes. Theoretical and Applied Genetics. 1999;98(2):186–94. doi: 10.1007/s001220051057

[2] Selvi A, Nair NV, Noyer JL, Singh NK, Balasundaram N, Bansal KC, et al AFLP analysis of the phenetic organization and genetic diversity in the sugarcane complex, Saccharum and Erianthus. Genet Resour Crop Ev. 2006;53(4):831–42. doi: 10.1007/s10722-004-6376-6

[3] Brown JS, Schnell RJ, Power EJ, Douglas SL, Kuhn DN. Analysis of clonal germplasm from five Saccharum species: S. barberi, S. robustum, S. officinarum, S. sinense and S. spontaneum. A study of inter- and intra species relationships using microsatellite markers. Genet Resour Crop Ev. 2007;54(3):627–48. doi: 10.1007/s10722-006-0035-z

[4] Heinz DJ. Sugarcane improvement through breeding. Developments in Crop Science. 1987.

[5] Shiba M, Kondo K, Miki E, Yamaji H, Morota T, Terabayashi S, et al Identification of medicinal Atractylodes based on ITS sequences of nrDNA. Biological & Pharmaceutical Bulletin. 2006;29(2):315–20. doi: 10.1248/bpb.29.315 16462038 10.1248/bpb.29.315

[6] Dkhar J, Kumaria S, Rao SR, Tandon P. Sequence characteristics and phylogenetic implications of the nrDNA internal transcribed spacers (ITS) in the genus Nymphaea with focus on some Indian representatives. Plant Systematics and Evolution. 2012;298(1):93–108. doi: 10.1007/s00606-011-0526-z

[7] Koloren O, Koloren Z, Eker Si. Molecular phylogeny of Artemisia species based on the internal transcribed spacer (ITS) of 18S-26S rDNA in Ordu Province of Turkey. Biotechnology & Biotechnological Equipment. 2016;30(5):1–6. doi: 10.1080/13102818.2016.1188674

[8] Clarkson JJ, Pennington TD, Chase MW, Haynes G, Engstrand R, Kaye M, et al Phylogenetic relationships in Trichilia (Meliaceae) based on ribosomal its sequences. Phytotaxa. 2016;259(1):6 doi: 10.11646/phytotaxa.259.1.4

[9] Yang CF, Yang LT, Li YR, Zhang GM, Zhang CY, Wang WZ. Sequence Characteristics and Phylogenetic Implications of the nrDNA Internal Transcribed Spacers (ITS) in Protospecies and Landraces of Sugarcane (Saccharum officinarum L.). Sugar Tech. 2016;18(1):8–15. doi: 10.1007/s12355-014-0355-9

[10] Liu XL, Li XJ, Liu HB, Xu CH, Lin XQ, Li CJ, et al Phylogenetic Analysis of Different Ploidy Saccharum spontaneum Based on rDNA-ITS Sequences. Plos One. 2016;11(3). doi: 10.1371/journal.pone.0151524 26986847 10.1371/journal.pone.0151524 PMC4795546

[11] Brookes AJ. The essence of SNPs. Gene. 1999;234(2):177–86. doi: 10.1016/S0378-1119(99)00219-X 10395891 10.1016/s0378-1119(99)00219-x

[12] Huang S, Deng L, Mei G, Li J, Lu K, Wang H, et al Identification of genome-wide single nucleotide polymorphisms in allopolyploid crop Brassica napus. BMC Genomics. 2013;14(1):717 doi: 10.1186/1471-2164-14-717 24138473 10.1186/1471-2164-14-717 PMC4046652

[13] Luo YM, Zhang WM, Ding XY, Shen J, Bao SL, Chu BH, et al SNP marker and allele-specific diagnostic PCR for authenticating herbs of Perilla. Acta Pharmaceutica Sinica. 2006;41(9):840–5. doi: 10.3321/j.issn:0513-4870.2006.09.008 17111830

[14] Szabo Z, Gyulai G, Humphreys M, Horvath L, Bittsanszky A, Lagler R, et al Genetic variation of melon (C-melo) compared to an extinct landrace from the Middle Ages (Hungary) I. rDNA, SSR and SNP analysis of 47 cultivars. Euphytica. 2005;146(1–2):87–94. doi: 10.1007/s10681-005-5685-y

[15] Ye S, Dhillon S, Ke X, Collins AR, Day INM. An efficient procedure for genotyping single nucleotide polymorphisms. Nucleic Acids Research. 2001;29(17):88–8. 11522844 10.1093/nar/29.17.e88 PMC55900

[16] Zhang SB, Xu FR, Zhang YH, Lin J, Song CF, Fang XW. Fine mapping and candidate gene analysis of a novel PANICLE AND SPIKELET DEGENERATION gene in rice. Euphytica. 2015;206(3):793–803. doi: 10.1007/s10681-015-1525-x

[17] Hou YP, Luo QQ, Chen CJ, Zhou MG. Application of tetra primer ARMS-PCR approach for detection of Fusarium graminearum genotypes with resistance to carbendazim. Australas Plant Path. 2013;42(1):73–8. doi: 10.1007/s13313-012-0162-2

[18] Garces-Claver A, Fellman SM, Gil-Ortega R, Jahn M, Arnedo-Andres MS. Identification, validation and survey of a single nucleotide polymorphism (SNP) associated with pungency in Capsicum spp. Theoretical and Applied Genetics. 2007;115(7):907–16. doi: 10.1007/s00122-007-0617-y 17882396 10.1007/s00122-007-0617-y

[19] Bacci M, Miranda VFO, Martins VG, Figueira AVO, Lemos MV, Pereira JO, et al A search for markers of sugarcane evolution. Genet Mol Biol. 2001;24(1–4):169–74. doi: 10.1590/S1415-47572001000100023

[20] Porebski S, Bailey LG, Baum BR. Modification of a CTAB DNA extraction protocol for plants containing high polysaccharide and polyphenol components. Plant Molecular Biology Reporter. 1997;15(1):8–15. doi: 10.1007/BF02772108

[21] Besse P. Nuclear Ribosomal RNA Genes: ITS Region. Methods Mol Biol. 2014;1115:141–9. doi: 10.1007/978-1-62703-767-9_7 24415473 10.1007/978-1-62703-767-9_7

[22] Medrano RFV, de Oliveira CA. Guidelines for the Tetra-Primer ARMS-PCR Technique Development. Mol Biotechnol. 2014;56(7):599–608. doi: 10.1007/s12033-014-9734-4 24519268 10.1007/s12033-014-9734-4

[23] Baldwin BG, Sanderson MJ, Porter JM, Wojciechowski MF. The its Region of Nuclear Ribosomal DNA: A Valuable Source of Evidence on Angiosperm Phylogeny. Annals of the Missouri Botanical Garden. 1995;82(2):247–77. doi: 10.2307/2399880

[24] Liu X, Zhang L, Li G, Qin R, Liu H. Application of nrDNA-ITS Sequences in Plant Phylogeny and Evolution. Botanical Research. 2014;3(1):32–40.

[25] Sang T, Crawford DJ, Stuessy TF. Documentation of reticulate evolution in peonies using internal transcribed spacer sequences of nuclear ribosomal DNA Implications for biogeography and concerted evolution. Proceedings Of The National Academy Of Sciences Of The United States Of America. 1995;92(15):6813–7. 7624325 10.1073/pnas.92.15.6813 PMC41419

[26] Waters ER, Schaal BA. Biased gene conversion is not occurring among rDNA repeats in the Brassica triangle. Genome. 1996;39(1):150–4. Epub 1996/02/01. doi: 10.1139/g96-020 . 18469883 10.1139/g96-020

[27] Qian J, Sun Y, Duan YH. Internal Transcribed Spacer Region of rDNA in Common Wheat and Its Genome Origins. Acta Agronomica Sinica. 2009;35(6):1021–9. doi: 10.1016/S1875-2780(08)60088-7

[28] Edme SJ, Glynn NG, Comstock JC. Genetic segregation of microsatellite markers in Saccharum officinarum and S. spontaneum. Heredity. 2006;97(5):366–75. doi: 10.1038/sj.hdy.6800888 16912699 10.1038/sj.hdy.6800888

[29] Mary S, Nair NV, Chaturvedi PK, Selvi A. Analysis of genetic diversity among Saccharum spontaneum L. From four geographical regions of India, using molecular markers. Genet Resour Crop Ev. 2006;53(6):1221–31. doi: 10.1007/s10722-005-2433-z

[30] Devarumath RM, Kalwade SB, Kawar PG, Sushir KV. Assessment of Genetic Diversity in Sugarcane Germplasm Using ISSR and SSR Markers. Sugar Tech. 2012;14(4):334–44. doi: 10.1007/s12355-012-0168-7

[31] Chandra A, Grisham MP, Pan YB. Allelic divergence and cultivar-specific SSR alleles revealed by capillary electrophoresis using fluorescence-labeled SSR markers in sugarcane. Genome. 2014;57(6):363–72. doi: 10.1139/gen-2014-0072 25247737 10.1139/gen-2014-0072

[32] Blattner FR. TOPO6: a nuclear single-copy gene for plant phylogenetic inference. Plant Systematics & Evolution. 2016;302(2):1–6. doi: 10.1007/s00606-015-1259-1

[33] Mccormack JE, Hird SM, Zellmer AJ, Carstens BC, Brumfield RT. Applications of next-generation sequencing to phylogeography and phylogenetics. Molecular Phylogenetics & Evolution. 2013;66(2):526–38. doi: 10.1016/j.ympev.2011.12.007 22197804 10.1016/j.ympev.2011.12.007

[34] Linder HP, Naciri Y. Species delimitation and relationships: The dance of the seven veils. Taxon. 2015;64(1):3–16. doi: 10.12705/641.24

[35] De SR, Adams KL, Vandepoele K, Van Montagu MC, Maere S, Van d P Y. Convergent gene loss following gene and genome duplications creates single-copy families in flowering plants. Proceedings of the National Academy of Sciences of the United States of America. 2013;110(8):2898–903. doi: 10.1073/pnas.1300127110 23382190 10.1073/pnas.1300127110 PMC3581894

[36] Piperidis G, Piperidis N, D’Hont A. Molecular cytogenetic investigation of chromosome composition and transmission in sugarcane. Mol Genet Genomics. 2010;284(1):65–73. doi: 10.1007/s00438-010-0546-3 20532565 10.1007/s00438-010-0546-3

[37] Wang P. Chromosome genetic analysis of nobilization of Saccharum spontaneum: Fujian Agriculture and Forestry University; 2016.

